# Comprehensive Characterization of Cachexia-Inducing Factors in Diffuse Large B-Cell Lymphoma Reveals a Molecular Subtype and a Prognosis-Related Signature

**DOI:** 10.3389/fcell.2021.648856

**Published:** 2021-05-17

**Authors:** Zhixing Kuang, Xun Li, Rongqiang Liu, Shaoxing Chen, Jiannan Tu

**Affiliations:** ^1^Department of Radiation Oncology, Nanping First Hospital Affiliated to Fujian Medical University, Nanping, China; ^2^Department of Oncology, Haikou Hospital Affiliated to Xiangya Medical College, Central South University, Haikou, China; ^3^Department of Radiation Oncology, Zhangzhou Hospital Affiliated to Fujian Medical University, Zhangzhou, China; ^4^Department of Oncology, Nanping First Hospital Affiliated to Fujian Medical University, Nanping, China

**Keywords:** cachexia-inducing factors, molecular subtype, prognosis-related, signature, diffuse large B-cell lymphoma

## Abstract

**Background:**

Cachexia is defined as an involuntary decrease in body weight, which can increase the risk of death in cancer patients and reduce the quality of life. Cachexia-inducing factors (CIFs) have been reported in colorectal cancer and pancreatic adenocarcinoma, but their value in diffuse large B-cell lymphoma (DLBCL) requires further genetic research.

**Methods:**

We used gene expression data from Gene Expression Omnibus to evaluate the expression landscape of 25 known CIFs in DLBCL patients and compared them with normal lymphoma tissues from two cohorts [GSE56315 (*n* = 88) and GSE12195 (*n* = 136)]. The mutational status of CIFs were also evaluated in The Cancer Genome Atlas database. Based on the expression profiles of 25 CIFs, a single exploratory dataset which was merged by the datasets of GSE10846 (*n* = 420) and GSE31312 (*n* = 498) were divided into two molecular subtypes by using the method of consensus clustering. Immune microenvironment between different subtypes were assessed *via* single-sample gene set enrichment analysis and the CIBERSORT algorithm. The treatment response of commonly used chemotherapeutic drugs was predicted and gene set variation analysis was utilized to reveal the divergence in activated pathways for distinct subtypes. A risk signature was derived by univariate Cox regression and LASSO regression in the merged dataset (*n* = 882), and two independent cohorts [GSE87371 (*n* = 221) and GSE32918 (*n* = 244)] were used for validation, respectively.

**Results:**

Clustering analysis with CIFs further divided the cases into two molecular subtypes (cluster A and cluster B) associated with distinct prognosis, immunological landscape, chemosensitivity, and biological process. A risk-prognostic signature based on CCL2, CSF2, IL15, IL17A, IL4, TGFA, and TNFSF10 for DLBCL was developed, and significant differences in overall survival analysis were found between the low- and high-risk groups in the training dataset and another two independent validation datasets. Multivariate regression showed that the risk signature was an independently prognostic factor in contrast to other clinical characteristics.

**Conclusion:**

This study demonstrated that CIFs further contribute to the observed heterogeneity of DLBCL, and molecular classification and a risk signature based on CIFs are both promising tools for prognostic stratification, which may provide important clues for precision medicine and tumor-targeted therapy.

## Introduction

Diffuse large B-cell lymphoma (DLBCL) is a biologically and clinically heterogeneous B-cell neoplasm morphologically characterized by large lymphoid cells with B-cell markers growing in a rapidly proliferating and diffuse pattern (Caimi et al., 2016). DLBCL is one major subtype of non-Hodgkin lymphoma (NHL) which originates from B-cells, and it constitutes more than 25–35% of NHL cases in developing countries (Miao et al., 2019). It is estimated that 81,560 people in the United States will be diagnosed with NHL, and 20,720 of those will die of related causes in 2021 (Siegel et al., 2021). In the last decades, dramatic improvements have been achieved in the treatment of DLBCL, and the regimen of rituximab, cyclophosphamide, doxorubicin, vincristine, and prednisone (R-CHOP) has been established as the first-line or standard therapy for patients diagnosed with DLBCL. Approximately 60% of cases can be cured by using this treatment strategy (Sarkozy et al., 2015). However, in the light of huge heterogeneity in all patients, more than one-third of individuals will fail this first-line therapy and experience extremely poor prognosis (Voltin et al., 2020), illustrating the unmet need to emphasize the importance of risk stratification that can lead to more scientific and effective personalized treatment. In recent times, the risk assessment of DLBCL has mainly concentrated on the international prognostic index (IPI) and cell of origin (COO); the application of COO classification in DLBCL has revealed two subtypes, namely, the germinal center B-cell-like (GCB) and activated B-cell-like (ABC) (Moffitt and Dave, 2017) subtypes. However, both IPI and COO are widely questioned regarding the risk stratification of a small number of DLBCL and do not accurately predict the outcome for cases (Wight et al., 2018) because the distinction based on COO does not fully account for the heterogeneous outcomes and chemotherapy response of DLBCL. The recent improvement in bioinformatics algorithm and microarray technology provided huge opportunities for clinical applications of paraffin-embedded tissue and brings a new dawn to the risk classification of DLBCL. The non-negative matrix factorization consensus clustering algorithm used by Chapuy et al. (2018) and the GenClass algorithm were employed by Schmitz et al. (2018) to analyze the genetic data of 304 and 574 cases of patients with DLBCL, respectively. Their analyses showed the existence of distinct subtypes independent of or within the COO subtypes. According to these previously reported studies, we hypothesized that the analysis of a gene expression signature may add considerable texture to improve the classification for risk stratification and personalized therapeutic implication in DLBCL.

Cachexia is a non-specific symptom characterized by a state of involuntary substantial loss of skeletal muscle mass with or without adipose tissue loss and is usually difficult to rehabilitate by conventional nutritional support (Mallard et al., 2019). Cachexia severely compromises life quality and reduces treatment tolerance among patients with cancer and contributes to 20% of all cancer deaths (Fearon et al., 2012). Weight loss greater than 10% in 6 months is determined to be one of the B symptoms and has been confirmed in multiple large retrospective research as an adverse prognostic factor for NHL, independent of IPI (Han et al., 2013; O’Brian et al., 2016; Xiao et al., 2017; Wight et al., 2018). Patients with the same height and a similar tumor burden but with a different cachexia status will receive a completely different chemotherapy drug regimen and are typically associated with distinct prognoses. Several tumor-derived and inflammatory factors are classified as cachexia-inducing factors (CIFs) and are derived from the tumor secretome or host; these are suggested to be involved in the pathogenesis of patients and drive the development of cachexia (Pettersen et al., 2020). Thus far, several markers for cachexia, such as serum albumin, body mass index, adipopenia, and sarcopenia, have been investigated and suggested to be likely factors affecting the prognosis of DLBCL (Go et al., 2020). Furthermore, 25 known CIFs were reported in a previous study, and their prognosis value was explored in 12 cancer types except DLBCL (Freire et al., 2020); hence, appropriate attention should be paid to CIFs in the context of DLBCL.

In this study, we comprehensively analyzed and determined the potential prognostic value of the 25 CIFs in DLBCL and stratified 884 patients into two subtypes based on the expression levels of these 25 CIFs. Subsequently, a deeper characterization of the immune microenvironment and biological process of the two subtypes was conducted. In addition, treatment sensitivity of commonly used drugs was predicted for patients with a distinct subtype. Moreover, we developed a multi-CIFs-based signature by utilizing the LASSO Cox regression model to predict the overall survival (OS) of patients with DLBCL. The prognostic accuracy of this signature was validated in two independent cohorts. Our signature can complement the existing risk stratification systems including COO and IPI score for prediction of outcome in DLBCL, possibly enabling physicians to make more informed treatment decisions.

## Materials and Methods

### Dataset Sources and Selection as Well as Data Processing

The raw CEL data of GSE56315 (55 DLBCL samples and 33 normal B-cell samples), GSE12195 (73 DLBCL samples and 20 normal B-cell samples), GSE12453 [11 DLBCL samples, 25 normal B-cell samples, and 12 cases of classical Hodgkin’s lymphoma (cHL)], GSE10846 (420 cases of DLBCL), GSE31312 (498 cases of DLBCL), and GSE87371 (223 cases of DLBCL), all of which were based on the GPL570 platform (HG-U133_Plus_2), were selected and downloaded. GSE32918 (249 cases of DLBCL) based on the platform of GPL8432 (Illumina HumanRef-8 WG-DASL v3.0) was downloaded in the form of a preprocessed expression matrix uploaded by the authors. All datasets were extracted from the Gene Expression Omnibus (GEO)^1^ database. The selection criteria for DLBCL datasets were as follows: (a) all expression profiling datasets based on any platform except those based on HG-U133A platform (the HG-U133A platform was developed 20 years ago, and the number of probes is less than half of that of other platforms), (b) all datasets should have basic clinical data characteristics including sex, age, OS, and OS status, and (c) datasets with a larger sample size and the minimum number of patients being > 200.

Using these criteria, the DLBCL datasets of GSE10846, GSE31312, GSE87371, and GSE32918 were identified and used to perform prognostic analysis. All the raw chip data went through the process of quality assessment, quality control, background correction, and normalization, and the process was completed by “simpleaffy” (version 2.64.0), “affyPLM” (version 1.64.0), and “arrayQualityMetrics” (version 3.46.0) packages. All microarray data were converted into expression matrix after processing. Finally, 1,529 cases of DLBCL, 78 cases of normal B-cell tissue, and 12 cases of cHL were included in the GEO dataset. All samples that lacked survival information and/or had survival data of < 1 day were excluded from further analysis.

### Landscape of Expression and Genetic Variation as Well as Prognostic Value of CIFs in DLBCL

To clarify the expression difference of 25 CIFs (CCL2, CD40LG, CSF1, CSF2, CSF3, CXCL12, CXCL8, FGF2, HGF, IFNG, IL10, IL15, IL17A, IL1B, IL4, IL6, LEP, LIF, MMP13, PDGFB, TGFA, TNF, TNFSF10, TNFSF11, and VEGFA) between DLBCL and normal B-cell tissues and to ensure its reliability, ANOVA was performed to calculate the discrimination in the two datasets, namely, GSE56315 and GSE12195. Based on the expression value of the 25 CIFs, principal component analysis was also performed to assess the distribution between the DLBCL and normal B-cell tissues. The mutation status and influence of mutation status on the survival of all CIFs in 48 cases of DLBCL patients was obtained from the cBioPortal database^2^. The samples with complete survival data in GSE10846 and GSE31312 were merged into a single meta-cohort (*N* = 882), and combat algorithm of “sva” package (version 3.38.0) was used to combine the datasets and remove batch effects to reduce non-biological technical biases. Genomic instability often generates a diversity of genome, leads to cancer occurrence, and influences disease development. Thus, the presence of deletions and accumulation of amplifications of CIFs were investigated. A univariate Cox regression model was adopted to calculate the hazard ratios (HRs) for each CIF in DLBCL patients, and Pearson’s correlation analysis was utilized to evaluate the positive or negative regulatory relationship among the 25 CIFs. The network of related relationships of a CIF whose value of expression was correlated with one or more CIFs (| Pearson *R*| > 0.1 and *P* < 0.001) was visualized by Cytoscape software (version 3.8.2).

### Unsupervised Clustering for 25 CIFs in DLBCL

Unsupervised clustering analysis was employed to detect unknown possible distinct subtypes based on the expression of 25 CIFs and differentiated in the meta-cohort (*n* = 882) for further analysis. The consensus cluster algorithm was performed by “ConsensuClusterPlus” package (version 1.52.0) to determine the number of clusters and stability of classification, and 1,000 repetitions were conducted to ensure the accuracy of the results (Wilkerson and Hayes, 2010). To determine the influence of distinct subtype on prognosis, Kaplan–Meier analysis was conducted and compared by log-rank test, and Kruskal–Wallis test was utilized to distinguish the expression of CIFs between different subtypes.

### Estimation of Immune Infiltration and Prediction of Cytotoxic and Immunomodulator Drug Sensitivity

To gain deeper insights into the tumor microenvironment of patients with DLBCL, CIBERSORT was used to calculate the composition difference of 22 kinds of infiltrating immune cells in DLBCL and normal B-cells. *P* < 0.05 was considered to indicate statistical significance. In addition, although the remarkable outcome of anti-PD-1 therapy in classic Hodgkin’s lymphoma (cHL) is acknowledged, the efficacy of anti-PD-1 monotherapy in DLBCL remains unsatisfactory and needs further investigation (Kline et al., 2020). Therefore, the distribution of immune cells in the microenvironment of cHL and DLBCL was also calculated. Single-sample gene set enrichment analysis (ssGSEA) algorithm which is based on 29 immune gene sets was applied to comprehensively quantify the relative abundance of immune cell types, pathways, functions, and checkpoints in each patient. The difference of 29 immune gene sets and 22 immune cells between cluster A and cluster B patients was analyzed using Kruskal–Wallis testing. In addition, the “pRRophetic” package (version 0.5) (Geeleher et al., 2014) was utilized to predict the treatment response for cytotoxicity and molecular targeted therapy between patients in cluster A and those in cluster B to determine their sensitivity to commonly used drugs for DLBCL.

### Gene Set Variation Analysis and Functional Annotation

To provide deeper insights into the heterogeneity of biological processes between cluster B and cluster A patients, gene set variation analysis (GSVA) enrichment analysis was performed by using “GSVA” R packages (version 1.36.3). GSVA is a non-parametric unsupervised analysis method mainly employed in expression dataset and is widely used to evaluate the variation in biological process activity and pathway in the samples of an expression dataset. The gene sets of “c2.cp.kegg.v6.2.symbols.gmt” were selected and downloaded from MSigDB database^3^ for implementing the GSVA analysis. Only adjusted *P* < 0.05 values were considered as statistically significant. Moreover, the “limma” package (version 3.44.3) was utilized to determine different biological pathways between cluster A and cluster B patients, and the results of |log2(fold change)| > 0.2 and *P* < 0.05 were considered to be statistically significant (Song et al., 2020). In addition, the R package of “limma” (version 3.44.3) was used to identify differentially expressed genes (DEGs) between cluster B and cluster A with the criterion of |log2(fold change)| > 1 and *P* < 0.05 for Gene Ontology (GO) and pathway enrichment analysis.

### Generation and Validation of Prognostic Signature Based on CIFs

Univariate Cox proportional hazard regression analysis was utilized to assess the relationship between CIFs and OS of DLBCL patients within the meta-cohort (which was incorporated by GSE10846 and GSE31312, and the meta-cohort was set as the training group). Only *P* < 0.05 was considered to indicate the most valuable prognostic CIF genes which were sorted out to perform the LASSO Cox regression analysis which depend on the R package “glmnet” (version 4.1). LASSO Cox regression analysis is a well-established and widely used mathematical selection method for screening the most predictive markers. The most prominent advantage of LASSO Cox regression is that, by penalized regression on all variable coefficients, the relatively unimportant coefficients of independent variables whose coefficients are close to 0 are excluded from the model. The optimal values of the penalty parameter *λ* were determined through 10 cross-validations. The following formula was derived to calculate the risk score based on the expression of candidate CIF genes, weighted by the regression coefficient obtained from LASSO Cox regression analysis in the training dataset:

$$\mathrm{Risk}\,\mathrm{score}=\sum_{i=1}^{n}e\,x\,p_{i}\times \beta_{i}$$

where *i* is the number of CIF genes, *exp*_*i*_ represents the expression value of CIF gene *i*, and β*i* represents the regression coefficient. By setting the median risk score as the cutoff value, all DLBCL patients were dichotomized into high- and low-risk groups. To evaluate the stability and reproducibility of the CIF signature, two external datasets including GSE87371 (*n* = 221) and GSE32918 (*n* = 244) were validated. Survival curves were constructed using the Kaplan–Meier method and carried out using the “survival” package in R (version 3.2-7). In addition, we used the “medcalc” statistical software to evaluate the performance of our CIF signature for its ability to discriminate molecular subtype with poor prognosis in DLBCL patients who were recently identified.

### Comprehensive Analysis of Risk Stratification and Clinical Attributes

To investigate the effect of the CIF-based risk signature on the prognosis of DLBCL, univariate and multivariate Cox regression analyses were conducted. The risk signature and other clinicopathological attributes including sex, age, stage, COO type, extranodal sites involved, serum LDH level, IPI score, bulky disease, B-symptoms, and Eastern Cooperative Oncology Group (ECOG) performance were entered into the analysis. All clinicopathological parameters were grouped according to the IPI criteria: serum LDH level, >1 × normal; ECOG performance status, ≥2; extranodal sites involved, >1; age, >60 years; and Ann Arbor stage, III–IV. All other statistical analyses were conducted using R (version 4.0.2).

## Results

### Patient Characteristics

A total of 1,475 patients with DLBCL and 53 with normal B cells from six independent academic institutions were included in the analysis after excluding samples that lacked clinical metadata; of these, 1,347 DLBCL samples from four datasets with survival time were used for prognosis-related research. The clinical characteristics of the 1,347 patients are presented in Table 1 and Supplementary Table 1. The median follow-up was 28.62 months [interquartile range (IQR): 11.22–52.14] for patients in the GSE10846 cohort, 34.32 months (17.25–55.42) for those in the GSE31312 cohort, 39.84 months (4.10–70.8) for those in the GSE32918 cohort, and 35.49 months (22.53–49.31) for those in the GSE87371.

**TABLE 1:** Clinical characteristics of the 1,347 cases of diffuse large B-cell lymphoma patients.

**Characteristics**	**GSE10846**	**GSE31312**	**GSE32918**	**GSE37371**
	**(*n* = 412)**	**(*n* = 470)**	**(*n* = 244)**	**(*n* = 221)**
**Age**				
< 60	179 (43.4%)	186 (39.6%)	68 (27.9%)	109 (49.3%)
≥ 60	233 (56.6%)	284 (60.4%)	176 (72.1%)	112 (50.7%)
**Sex**				
Male	222 (53.9%)	271 (57.7%)	142 (58.2%)	116 (52.5%)
Female	172 (41.8%)	199 (42.3%)	102 (41.8%)	105 (47.5%)
NA	18 (4.3%)			
**Stage**				
I–II	188 (45.6%)	223 (47.4%)		71 (32.1%)
III–IV	217 (52.7%)	247 (52.6%)		150 (67.9%)
NA	7 (1.7%)			
**COO type**				
GCB	182 (44.2%)	230 (48.9%)	119 (48.8%)	82 (37.1%)
ABC	167 (40.5%)	197 (41.9%)	79 (32.4%)	85 (38.5%)
Unclassified	63 (15.3%)	43 (10.2%)		54 (24.4%)
NA			46 (18.8%)	
**ECOG performance**				
0–1	295 (71.6%)	374 (79.6%)		
2–4	93 (22.6%)	96 (20.4%)		
NA	24 (5.8%)			
**LDH escalated**				
Yes	177 (43.0%)	275 (58.5%)		
No	173 (42.0%)	149 (31.7%)		
NA	62 (15.0%)	46 (9.8%)		
**Bulky**				
Yes		94 (20.0%)		
No		271 (57.7%)		
NA		105 (22.3 %)		
**IPI score**				
0–2		275 (58.5%)		119 (53.8%)
3–5		148 (31.5%)		102 (46.2%)
NA		47 (10.0%)		
**B symptom**				
Yes		130 (27.7%)		
No		278 (59.1%)		
NA		62 (13.2%)		
**Extranodal sites**				
Yes	145 (35.2%)	278 (59.1%)		
No	236 (57.3%)	192 (40.9%)		
NA	31 (7.5%)			

### Cachexia-Inducing Factors Are Up-Regulated in DLBCL

**FIGURE 1:**
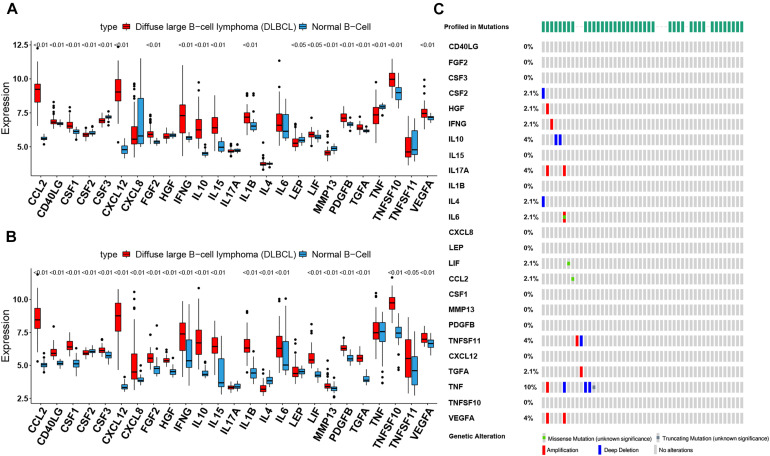
The landscape of cachexia-inducing factors in the diffuse large B-cell lymphoma (DLBCL). **(A,B)** Expression levels of 25 cachexia-inducing factors in DLBCL and normal B cell from human tonsils (**A**, GSE56315; **B**, GSE12195). The black dots represent outliers. **(C)** The mutation frequency of 25 CIFs in 48 patients with DLBCL from The Cancer Genome Atlas cohort.

**FIGURE 2:**
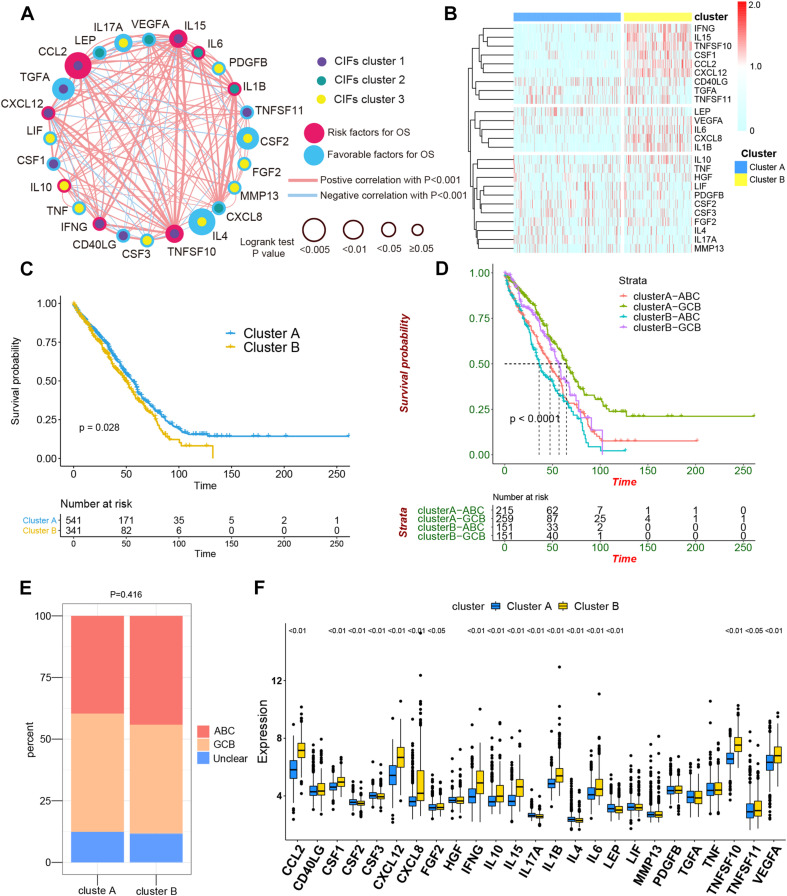
The comprehensive landscape of cachexia-inducing factor (CIF) interactions and identification of two molecular subtypes with different prognoses and transcriptome traits. **(A)** Network showing the landscape of CIF interactions and their prognostic significance for patients with DLBCL. The circle size represented the effect of each CIF on the prognosis, and the range of values calculated by log-rank test was *p* < 0.005, *p* < 0.01, *p* < 0.05, and *P* ≥ 0.05, respectively. Red circle, risk factors of prognosis. Blue circle, protective factors of prognosis. The lines linking the CIFs showed their interactions, and the thickness of the connecting line is positively correlated with the strength of the correlation. Negative correlation was marked with blue and positive correlation with red. Dots in the circle represent three CIF gene clusters termed as CIF clusters 1–3 and marked with purple, dark cyan, and yellow, respectively. **(B)** Heat maps showing the 25 CIFs’ expression level clustered by different subtypes and segregation according to the relevance of CIFs. **(C)** Survival analyses for the two molecular subtypes based on 882 patients with diffuse large B-cell lymphoma (DLBCL) from two Gene Expression Omnibus cohorts (GSE10846 and GSE31312) including 541 cases in cluster A and 341 cases in cluster B. Kaplan–Meier curves with log-rank *p* value 0.028 showed a significant survival difference among distinct subtypes. **(D)** Patients separated by cell of origin (COO) subtype with distinct molecular subtypes have a significantly different prognosis. **(E)** The proportion of COO subtypes in cluster A and cluster B patients. **(F)** Difference in the expression of 25 CIFs between cluster A and cluster B subtype groups.

To assess the biological function of CIFs, the expression profiles of 25 CIFs in two cohorts were obtained for systematically investigating the distinct expression patterns between DLBCL and normal B-cell tissues. Almost all CIFs were dramatically over-expressed in DLBCL that comprised the dataset GSE56315, which was subsequently validated in another dataset, GSE12195. Nineteen CIFs were identified to be up-regulated in GSE56315 and 21 CIFs were over-expressed in GSE12195 (Figures 1A,B). *CCL2*, *CD40LG*, *CSF1*, *CSF3*, *CXCL12*, *FGF2*, *IFNG*, *IL10*, *IL15*, *IL1B*, *LIF*, *MMP13*, *PDGFB*, *TGFA*, *TNFSF10*, and *VEGFA* were all up-regulated in both datasets, except for CSF2 which was down-regulated in DLBCL. Furthermore, the expression level of IL17A showed no statistically significant difference between the DLBCL and normal B cell tissues (*P* > 0.05). Based on the expression level of these 25 CIFs, we could accurately distinguish DLBCL from normal samples (Supplementary Figures 1A,B). The high heterogeneity of the expression landscape indicated that CIFs play an essential biological role in DLBCL pathogenesis and progression. Apart from this, we first summarized somatic mutations of the 25 CIFs in DLBCL patients based on The Cancer Genome Atlas cohort. Thirteen CIFs were found with experienced mutations, and TNF showed the highest frequency of mutations followed by *VEGFA*, *IL17*, and *IL10* (Figure 1C). In addition, patients with *LIF* and *TGFA* mutations showed a negative correlation with survival (Supplementary Table 2). Three CIF gene clusters were identified by unsupervised clustering analysis (Figure 2B), and most CIFs in the same cluster had a positive regulatory relationship with each other except in CIF cluster 3 (Figure 2A and Supplementary Table 3). A univariate Cox regression model was also designed to reveal the prognostic value of 25 CIFs in DLBCL patients of the meta-cohort that was enrolled by two GEO datasets (GSE10846 and GSE31312) after batch correction (Supplementary Figures 2A–D), and seven CIFs (*CCL2*, *CSF2*, *IL15*, *IL17A*, *IL4*, *TGFA*, and *TNFSF10*) were significantly associated with OS (Figure 2A and Supplementary Table 4). The comprehensive landscape of CIF interactions and their prognostic significance for patients with DLBCL were delineated with the network (Figure 2A).

### Consensus Clustering for CIFs and Identifying Molecular Subtypes of DLBCL

All in all, 882 cases of DLBCL from the meta-cohort (*n* = 882) were utilized to find a stable and reliable subtype classification at the end of the repeat sampling. Thus, *k* = 2 was identified as the optimal number of clustering based on the expression levels of CIFs and the result of proportion of ambiguous clustering (PAC) measure (Supplementary Figures 3A–H). A total of 882 DLBCL patients were clustered into two subtypes named as cluster A (*n* = 541) and cluster B (*n* = 341) (Supplementary Table 5). Cluster B was significantly associated with poor OS, and the 50-month OS rates for cluster A and cluster B patients were 31.6 and 24.0% (Figure 2C). Integration of consensus clustering and COO-based classification from the 882 patients and Kaplan–Meier curves also showed that patients separated by COO with distinct molecular signature had a significantly different prognosis (*p* < 0.0001, Figure 2D). The ABC of COO subtypes accounts for a larger population in cluster A than in cluster B (Figure 2E), but there were no significant differences (*p* = 0.416). As expected, an increased expression of most CIFs was observed in high-risk cases with DLBCL (Figure 2F), and the variation of CIF expression in different molecular subtypes further showed heterogeneity of DLBCL.

### Distinct Immune Cell Infiltration and Molecular Function Between Different Molecular Subtypes

CIBERSORT immune analysis confirmed that DLBCL was associated with decreased naive B cells and memory B cells and had an abundance of activated memory CD4 T cells, follicular helper T cells, M0 macrophages, M1 macrophage, and M2 macrophages in three independent cohorts (Supplementary Figures 4A–C). However, DLBCL showed higher infiltration levels of CD8 T cells and lower expression of CD274 (PD-L1) than cHL (Supplementary Figure 4C).

**FIGURE 3:**
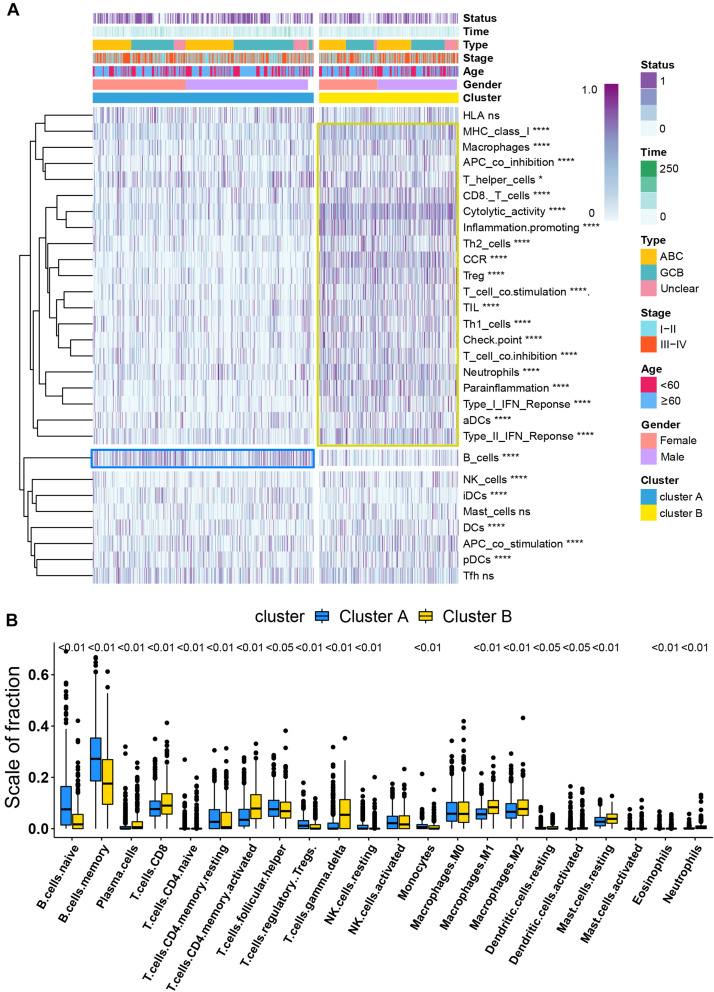
Immune signature analysis. **(A)** Unsupervised hierarchical clustering of immune cell types and functions by individual subtypes (cluster A, blue; cluster B, yellow. *P < 0.05; **P < 0.01; ***P < 0.001; and ****P < 0.0001). **(B)** Comparative fraction of the immune cell infiltration between cluster A and cluster B subtypes.

**FIGURE 4:**
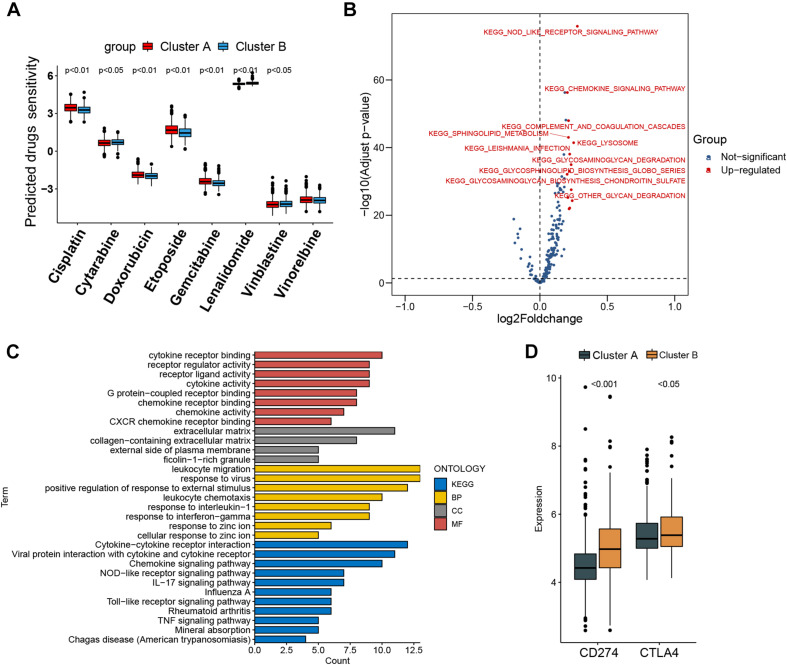
Prediction of chemotherapy and immunomodulatory effect and biological characteristics in distinct subtypes. **(A)** Sensitivity analysis of eight common therapeutic drugs in patients of cluster A and cluster B. **(B)** Differences in pathway activities scored by gene set variation analysis between cluster A and cluster B patients. Red dot indicates activated pathways in cluster B patients, and blue dot indicates insignificant activated pathways between cluster A and cluster B patients. **(C)** Functional annotation and Kyoto Encyclopedia of Genes and Genomes pathway enrichment analysis for differentially expressed genes between cluster A and cluster B patients. BP, biological process; CC, cellular component; MF, molecular function. **(D)** CD274 (PD-L1) and CTLA4 expression difference in cluster A and cluster B.

Per recent findings of distinct prognosis between cluster A and cluster B, ssGSEA and CIBERSORT were used to define the distribution of immune landscape and pattern between the two subtypes, and the result showed that cluster A and cluster B have significant divergence in almost all components of immune cell types and immune functions. ssGSEA revealed that patients in cluster B were associated with a remarkably high number of activated dendritic cells (aDCs), APC co-inhibition, APC co-stimulation, cytokine and cytokine receptor (CCR), CD8+ T-cells, check-point, cytolytic activity, activated dendritic cells (DCs), immature dendritic cells (iDCs), inflammation promotion, macrophages, para-inflammation, NK cells, MHC class I, neutrophils, plasmacytoid dendritic cells (pDCs), T-cell co-inhibition, T-cell co-stimulation, T helper cells, Th1 cells (T helper 1), Th2 cells, tumor-infiltrating lymphocytes (TIL), regulatory T cells (Treg), type I IFN response, and type II IFN response. In comparison, cluster A patients showed a significantly high number of B cells. Unsupervised hierarchical clustering of immune cell types and functions are described in Figure 3A and Supplementary Table 6. CIBERSORT immune analysis also confirmed that cluster A showed an overrepresentation of naive B cell and memory B cells, whereas cluster B showed higher infiltration levels of CD8 T cells, plasma cells, CD4 T cells, CD4 naive T cells, activated memory T cells, gamma delta resting NK cells, activated NK cells, monocytes, M1 macrophages, eosinophils, M2 macrophages, resting dendritic cells, neutrophils, activated mast cells, activated dendritic cells, and resting mast cells (Figure 3B and Supplementary Table 7). Interestingly, the considerable inconsistencies in the scale of fraction of B cells between cluster A and cluster B patients presented in CIBERSORT were very similar to the results obtained in the ssGSEA analysis, indicating that the high proportion of B cells was associated with prolonged survival. To better illustrate the characteristics of immune cell infiltration and molecular function, we tested the correlation between immune cell infiltration obtained from CIBERSORT and immune landscape and molecular pattern acquired from ssGSEA (Supplementary Figure 5). In addition, *PD-L1* and *CTLA-4* were also identified as being considerably overexpressed in cluster B (Figure 4D).

### Heterogeneity of Drug Sensitivity and Biological Behaviors Between Different Molecular Subtypes

The IC50 of seven commonly used cytotoxic drugs (cisplatin, cytarabine, doxorubicin, etoposide, gemcitabine, vinblastine, and vinorelbine) and one immunomodulator drug (lenalidomide) was predicted for cluster B and cluster A patients (Supplementary Table 8). We found that cisplatin, doxorubicin, and etoposide had lower IC50 in cluster B patients, contrary to the result of cytarabine, vinblastine, and lenalidomide in cluster B patients (Figure 4A). Furthermore, to explore the discrepancy of biological behaviors between cluster A and cluster B, GSVA and GO as well as Kyoto Encyclopedia of Genes and Genomes (KEGG) pathway enrichment analysis were performed. As shown in Figure 4B and Supplementary Tables 9, 10, cluster B patients had markedly enriched pathways of NOD-like receptor signaling, chemokine signaling, cytokine–cytokine receptor interaction, hematopoietic cell lineage, and complement and coagulation cascades. Briefly, 79 DEGs were identified between cluster B and cluster A (Supplementary Figures 6A,B), and these DEGs were remarkably related to cytokine activity and cytokine-related pathway (Figure 4C and Supplementary Figures 7A–D), which re-confirmed that cytokine activity and cytokine-related pathway played a nonnegligible role in immune regulation in the tumor microenvironment.

### The Risk Signature Robustly Identifies DLBCL Patients With Poor Survival

**FIGURE 5:**
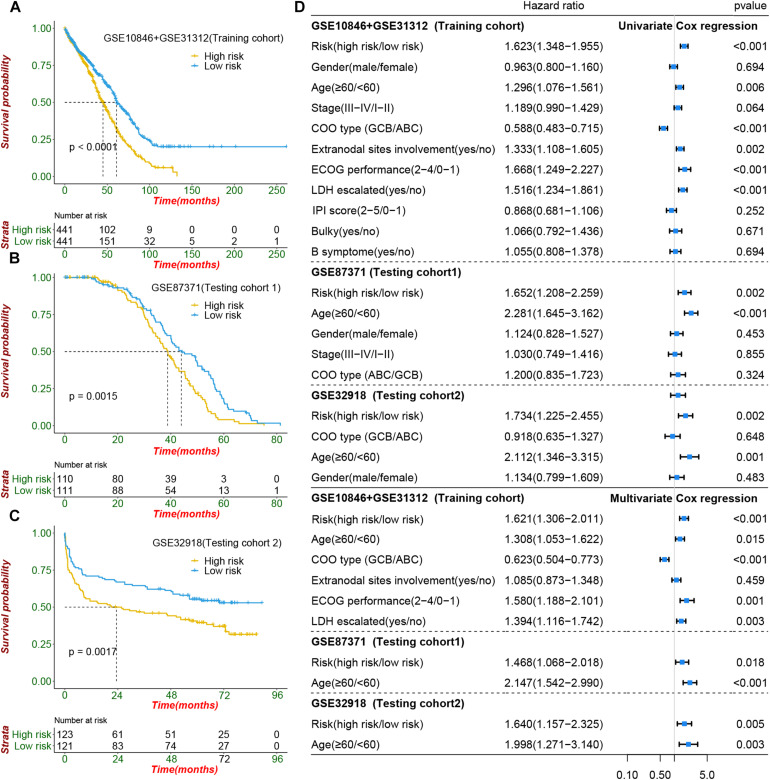
Prognostic value of cachexia-inducing factor (CIF) risk signature in patients with DIBCL. **(A–C)** Performance of the CIFs based on the risk signature in predicting overall survival in the training cohort and two independent testing cohorts. **(D)** Forest plot showing that the signature is significantly associated with prognosis and works independently of the cell of origin subtyping and all clinical features in univariate CoxPH and multivariate CoxPH analyses.

To construct a prognostic signature, seven CIFs that were identified as being associated with OS in the univariate Cox regression were included in the LASSO Cox regression model in the training dataset (882 samples selected from the meta-cohort). The optimal tuning parameter identified the following seven CIFs: CCL2, CSF2, IL15, IL17A, IL4, TGFA, and TNFSF10 (Supplementary Figures 8A,B). A risk score was then computed for each DLBCL patient based on the individual expression of the seven CIFs, weighted by the regression coefficient in the training set based on the following formula: risk score = (0.0668 × CCL2 expression) + (−0.2463 × CSF2 expression) + (0.05391 × IL15 expression) + (-0.2381 × IL17A expression) + (-0.2305 × IL4 expression) + (-0.1621 × TGFA expression) + (-0.1621 × TNFSF10 expression). Taking the median risk score as the cutoff value, all patients were divided into high- and low-risk groups. High-risk patients had a worse prognosis than low-risk ones [HR: 1.623 (1.348–1.995); *P* < 0.001] (Figure 5A). In addition to predicting survival, the performance of our risk signature to identify the cluster B molecular subtype recently identified with poor prognosis was determined, and it yielded an area under the curve value of 0.786 [95%CI (0.758–0.813); *P* < 0.001; Supplementary Figure 9A]. It showed that the distribution of risk scores between cluster A and cluster B vary significantly (*P* = 2.22e 10^–12^, Supplementary Figure 9B) and a large proportion (264 of 341, 77.42%) of patients in cluster B were classified into a high-risk group (Supplementary Figure 9C). The role of the risk signature was validated by an additional two datasets that were consistent with the initial findings of the training dataset. There was significant distinction in OS between the high- and low-risk patients, and patients who were categorized into the high-risk group had shorter OS than those categorized into the low-risk group, cohort-1 [GSE87371; HR: 1.652 (1.208–2.259); *P* = 0.002] and cohort-2 [GSE32918; HR: 1.734 (1.225–2.455); *P* = 0.002] (Figures 5B,C). Kaplan–Meier curves also showed that patients separated by distinct pathological type have a significantly different prognosis (Supplementary Figures 10A–C).

### The CIF Risk Signature Serves as an Independent Predictor of Risk and Survival Outcomes in DLBCL Patients

To evaluate whether the risk signature had an additional prognostic value that was beyond the clinical characteristics, univariate and multivariate Cox regression analyses were performed by clinical features and risk signature. In the univariate Cox regression, the seven-CIF-based risk signature was significantly correlated with OS. After multivariable adjustment by age, stage, COO type, extranodal sites involved, serum LDH level, and ECOG performance, the seven-CIF-based risk signature remained a powerful and independent prognostic factor for DLBCL patients (HR: 1.621, 95%CI: 1.306–2.011, *P* < 0.0001). Similar results were also noted in the testing cohort-1 dataset (HR: 1.468, 1.068–2.018; *P* = 0.018) as well as in the testing cohort-2 dataset (HR: 1.640, 1.157–2.325; *P* = 0.005) (Figure 5D). The observations in our study demonstrate that the CIF-based risk signature contributes to the additive prognostic value beyond that of age, pathological type, extranodal sites involved, serum LDH level, and ECOG in DLBCL patients.

## Discussion

Molecular classification of human cancers dividing patients into distinct molecular subtypes has unlocked an innovative approach to personalized medicine. Although the COO classification of GCB and ABC subtypes has been widely utilized to discriminate cells of DLBCL to predict patient prognosis, it is still debatable and considered unable to comprehensively demonstrate the distinct genetic and genomic characteristics of all DLBCLs (Wright et al., 2020). The extreme molecular heterogeneity of DLBCL brings a huge challenge to the development of precision treatment. Continuous progress in identification and differentiation of subtypes or risk stratification is needed to accelerate the management of personalized treatment in DLBCL. Cachexia is reportedly related to standard R-CHOP chemotherapy intolerance and significantly associated with a poor prognosis in DLBCL patients (Go et al., 2016). In the present study, we profiled the genomic landscape of CIFs in 882 DLBCL patients and revealed two distinct molecular subtypes with significantly different survival outcome and distinctive immune landscape, which captures the previously unexplained heterogeneity of the tumor microenvironment in DLBCL and may provide deeper insights into the heterogeneous responses to cytotoxic and immune blockade therapy. In addition, it may enable the development of subtype-specific treatment strategies targeting unique immune (therapeutic) vulnerabilities. Moreover, we developed and validated a seven-CIF-based risk signature to complement the existing prognostic evaluation system for the prediction of DLBCL outcome. To the best of our knowledge, this is the first study to comprehensively characterize the genomic landscape prognostic significance of CIFs in patients with DLBCL.

The molecular heterogeneity of DLBCL constitutes a major obstacle in treatment management of patients (Alkodsi et al., 2019). Significant efforts have been invested in molecular biology, and gene microarray technology has yielded significant public and invaluable gene expression data sets, and those data can be used for cancer or lymphoma risk stratification and pave the way for accurate disease classification (Li et al., 2017; Tang et al., 2018). To date, various molecular classification systems or mathematical clustering methods have been previously proposed; however, these classification approaches have their limitations and need further improvements. An unsupervised clustering of 2,118 genes’ expression analysis performed by Monti et al. identified three distinct subtypes of DLBCL, but the subtypes identified in this study were not associated with prognosis (Monti et al., 2005). By utilizing the method of recursive feature elimination support vector machine, Risueño et al. (2020) identified two subtypes in the GSE10846 dataset. Unfortunately, there is no significant difference in survival between the two subtypes. Karen Dybkaer et al. divided 1,139 samples of DLBCL into four genetic subtypes and evaluated the prognostic difference of those subtypes; it was seen that only the subclass of GCB presented prognostic stratification (Dybkaer et al., 2015). Another clustering methodology utilized by George Wright et al. determined seven subtypes of DLBCL, but the significantly distinctive outcome was only observed within the ABC subtype (Wright et al., 2020). Alkodsi et al. reported four subtypes of DLBCL by clustering the expression of 36 somatic hypermutation (SHM) genes, and those subtypes had a distinct clinical outcome (Alkodsi et al., 2019). However, the detection of gene SHM is more expensive and complicated than RT-PCR assay, which were limitations to routine clinical application. In this study, we investigated the contribution of CIFs to heterogeneity of distinct prognosis, immunological landscape, chemosensitivity, and biological process in DLBCL and showed two molecular subtypes defined by CIF expression patterns, and our subtypes showed distinctive prognosis within each of the COO subtypes.

It is well known that the presence of immune and inflammatory cells contributes to modulate tumor growth and invasion in DLBCL (Ennishi et al., 2020; Solimando et al., 2020). Characterization of immune infiltration and immune functions between different molecular subtypes provides important insights into the clinical outcome heterogeneity and pathogenesis of DLBCL. The naïve B-cells, memory B-cells, and macrophages in our study were the most represented cell proportions within the microenvironment of DLBCL patients. Normally, naive B-cells experience the germinal center and differentiate into either memory B-cells or plasma cells (for response to infections and secretion of high-affinity antibodies) to play a key role in humoral immunity (Bakhshi and Georgel, 2020). However, malignant transformation of DLBCL forms the mature B cells, which also experienced the germinal center reaction (Pasqualucci and Dalla-Favera, 2018). This transformation may contribute to an excessive consumption of naïve B cells and reduce the production of mature B cells. However, the number of B cells always plays a core role in the immune network and is related to prolonged survival (Bindea et al., 2013), which is consistent with our results. Our analysis revealed that the proportions of naïve B and memory B cells in DLBCL are significantly lower than the normal control group and represented lower fractions in cluster B which was associated with a worse prognosis. GSVA, GO, and KEGG enrichment results showed that cluster B, which had an abundance almost the same as that of immune cells, was strongly associated with cytokine activity and the chemokine pathway. This phenomenon may be related to the fact that immune cells are capable of producing multiple types of cytokines and chemokines (Tamma et al., 2020).

Macrophages, including M1 and M2 types, are more conspicuous than any other immune cell except B cells in DLBCL, and the proportion of M2 type macrophages was higher than that of macrophages M1. M1 macrophages have an antitumor response against neoplastic cells. Conversely, M2 macrophages have a predominant role of promoting tumor growth and progression (Poles et al., 2019). Macrophages usually maintain a balanced state; if macrophages M2 predominate, the balance may shift to a pro-tumor microenvironment (Riihijarvi et al., 2015). CTLA-4 is expressed on regulatory T (Treg) cells and is believed to act as an immune checkpoint receptor, which contributes to the inhibition and exhaustion of T-cells, and has an additional role in promoting the proliferation and survival of B-cell lymphoma (Herrmann et al., 2017). In our study, the number of regulatory T (Treg) cells was higher in cluster B than in cluster A, in line with the expression level of CTLA-4. Aberrant PD-L1 expression also offered a key immune escape mechanism in B-cell lymphoproliferative disorders, and increased PD-L1/PD-1 expression confers an adverse prognosis in DLBCL (Vari et al., 2018). The low overall response rate of anti-PD-1 antibody in DLBCL was attributed, at least to some extent, to the low expression of PD-L1 (Autio et al., 2020). Blockade of the PD1/PD-L1 axis showed particularly potent responses in cHL patients, and an increased expression of PD-L1 was associated with treatment response (Xu-Monette et al., 2018). We found that the expression level of PDL-1 in DLBCL tissue was significantly higher than in normal tissues but significantly lower than in cHL, which may explain why the efficacy of immunotherapy in DLBCL patients is not as good as that in cHL. Meanwhile, DLBCL patients with a higher expression level of PD-L1 seem to show a correlation with an increased resistance to frontline therapy but always related to prolonged PFS if treated with anti-PD-1 antibody (El Hussein et al., 2020; Wang L. et al., 2020). In line with this, cluster B which was associated with worse prognosis showed a higher expression level of CD274/PD-L1 than cluster A. The above-mentioned results suggest that cluster B patients may benefit more from PD-1 blockade therapy than cluster A patients.

Compared with a single mRNA, microRNA, or miRNA, integrating multiple biomarkers into a single signature by LASSO Cox regression could substantially improve the value of prognosis prediction (Zhang et al., 2013). In the present study, we focused on CIFs and developed a seven-CIF-based signature to predict OS in DLBCL. Another interesting aspect of our signature is that it works independently of COO subtyping and all clinical features. Although the potential of a signature based on miRNA expression has previously been reported in the prognostic stratification of DLBCL, but it is limited by a small sample size and lacks an independent cohort to validate its reliability (Montes-Moreno et al., 2011). Investigation of the biological function of the seven CIFs included in our signature has been conducted in previous studies. Interleukin (IL)-4 has been confirmed to be elevated in HL and follicular lymphoma; moreover, IL-4 not only contributes to the abnormal proliferation of lymphoma cells but also prevents malignant lymphocytes from apoptosis (Kawakami et al., 2005; Carey et al., 2007; Calvo et al., 2008). Additionally, IL-17A has been reported to have a role in promoting tumor growth and metastasis, but it also exhibited anti-cancer ability and showed a positive function in improving response to adjuvant chemotherapy in bladder cancer and gastric cancer (Kulig et al., 2016; Wang et al., 2019; Wang Z. et al., 2020). Granulocyte-macrophage colony-stimulating factor 2 (CSF2) one of the sub-members of the CSF family, has the capability of jeopardizing antitumor function and has a positive role in immunosuppression; furthermore, it can also improve antitumor efficacy through modulating the infiltration of immune cells in the tumor microenvironment and is associated with prolonged prognosis (Huang et al., 2020). TGFA has been previously confirmed as a crucial oncogenic mediator and promotes tumor cell growth *via* the TGF-α/EGFR signaling pathway (Wu et al., 2016). TNFSF10 was found to be involved in promoting tumor proliferation in non-Hodgkin’s lymphoma by activating the NF-κB pathway (Agrusa et al., 2020). CCL2 was positively related to TNFSF10 in our study and involved in the proliferation and survival of hematological tumors (Rafei et al., 2011). IL-15 is a proinflammatory cytokine that contributes STAT activation by mediating JAK1 and JAK3 phosphorylation, leading to lymphoma cell growth and survival. Nonetheless, the antitumor capacity of IL-15 by improving NK-cell function on the hematological malignancies has also been documented (Mishra et al., 2014; Mao et al., 2016).

Limitations of the present study should be acknowledged. Firstly, it is a retrospective research instead of a prospective study. Secondly, subtype classification and prognostic signature should be further validated for its efficacy in more independently prospective population. Finally, additional genetic and experimental studies of CIFs are required to elucidate the carcinogenesis and progression mechanism in DLBCL.

## Conclusion

Our results show that CIFs further contribute to the observed heterogeneity of DLBCL, with specific tumor microenvironment features associated with disease progression and severity. Furthermore, a novel signature based on CIFs was identified and validated in multiple groups of patients, which allows robust risk stratification and may facilitate the implementation of individualized treatment for DLBCL patients with a different prognosis.

## Data Availability Statement

The original contributions presented in the study are included in the article/Supplementary Material, further inquiries can be directed to the corresponding authors.

## Author Contributions

ZK, JT, and XL contributed to the conception, design, and further drafts. ZK, XL, and SC contributed to the development of methodology, analysis, and interpretation of data. RL, ZK contributed to the construction of figures and writing of the original draft. XL and JT contributed to supervision. All authors reviewed the manuscript and approved the final version to be published.

## Conflict of Interest

The authors declare that the research was conducted in the absence of any commercial or financial relationships that could be construed as a potential conflict of interest.

## Footnotes

1 http://www.ncbi.nlm.nih.gov/geo/

2 https://www.cbioportal.org/datasets/

3 http://www.gsea-msigdb.org/gsea/msigdb/index.jsp

## Supplementary Material

Supplementary Figure 1 Principal component analysis for the expression profiles of 25 cachexia-inducing factors (CIFS) to distinguish diffuse large B-cell lymphoma (DLBCL) from normal samples in GSE12195 and GSE56315 cohorts: **(A,B)**. Two subgroups without intersection were identified, indicating the DLBCL, and normal samples were well distinguished based on the expression profiles of CIFs.

Supplementary Figure 2 Box plot of expression data before and after normalization. The x-axis presents the different cohorts, and the y-axis presents the expression value. **(A)** Data before and after normalization of the expression profiles of GSE10846 and GSE31312. **(B)** The Venn diagram for intersection of the probe set of GSE10846 and GSE31312. **(C)** Samples distribution of the two cohorts are significantly different before batch correction. **(D)** Samples distribution of the two cohorts after batch correction.

Supplementary Figure 3 Unsupervised clustering of 25 cachexia-inducing factors in 882 cases of patients with diffuse large B-cell lymphoma (DLBCL) to identify distinct molecular subtypes. **(A–F)** Consensus matrices of the DLBCL cohort for *k* = 2–7, allowing quick and accurate visualization of cluster boundaries. **(G)** Consensus clustering cumulative distribution function for *k* = 2 to 9. **(H)** Tracking plot showing the consensus cluster of items (in columns) at *k* = 2 to 9 (in rows).

Supplementary Figure 4 Difference in the abundance of immune cell infiltration and expression of Pd-1 among diffuse large B-cell lymphoma (DLBCL), normal B cell, and classic Hodgkin’s lymphoma (cHl). **(A,B)** The proportion of immune cell in DLBCL and normal B cell extracted from tonsil: **(A)** GSE56315 and **(B)** GSE12195). **(C)** The proportion of immune cell infiltration and expression level of PD-L1 among DLBCL, normal B cell, and cHL. ^∗^*P* < 0.05; ^∗∗^*P* < 0.01; ^∗∗∗^*P* < 0.001, ^****^*P* < 0.0001.

Supplementary Figure 5 Correlation of immune landscapes and immune cell infiltration. Positive correlation was marked with blue, and negative correlation was marked with yellow.

Supplementary Figure 6 Differentially expressed genes in cluster B and cluster a patients. **(A)** Heat map for differentially expressed genes in cluster B and cluster a patients. **(B)** Volcano plot of differentially expressed genes in cluster B and cluster a patients. Red, significantly upregulated genes; blue, significantly downregulated genes; Fc, fold change.

Supplementary Figure 7 Functional enrichment analysis for differentially expressed genes (DEGs) between cluster B and cluster a patients. **(A)** Kyoto Encyclopedia of Genes and Genomes analyses for DEGs. **(B)** Biological process. **(C)** Cellular component. **(D)** Molecular function.

Supplementary Figure 8 Identification of the risk signature by least absolute shrinkage and selection operator (Lasso) Cox regression. **(A)** Lasso coefficient of the seven cachexia-inducing factors associated with overall survival in univariate Cox regression. **(B)** Ten-fold cross-validation for tuning the parameter selection in the Lasso module.

Supplementary Figure 9 Performance of the cachexia-inducing factor (CIFs) signature in identifying poor molecular subtypes in the training cohort. **(A)** Comparative risk score between cluster a subtype and cluster B subtype. **(B)** Receiver operating characteristic curves to depict the accuracy of CIFs risk signature in identifying cluster B which was with poor prognosis. **(C)** Alluvial diagram showing the changes of CIFs cluster subtypes, risk, and status.

Supplementary Figure 10 Performance of combinations of the prognostic model and cell of origin subtype in the prediction of patients with DLBCL in the training cohort and two independent testing cohorts. **(A)** GSE10846 + GSE31312. **(B)** GSE87371. **(C)** GSE32918.

Supplementary Table 1 Clinical characteristics of the 882 cases of DLBCL patients which was merged by GSE10846 and GSE31312.

Supplementary Table 2 Prognostic analysis for mutation satatus of 13 CIFs in 48 cases of DLBCL.

Supplementary Table 3 Spearman correlation analysis of the 25 CIFs.

Supplementary Table 4 Prognostic analysis of 25 CIFs in 884 cases of DLBCL using a univariate Cox regression model.

Supplementary Table 5 consensus clustering analysisi results of 884 cases of DLBCL.

Supplementary Table 6 Estimating relative abundance of tumor microenvironment cells in 884 case of DLBCL patients by the Single-Sample Gene-Set Enrichment Analysis (ssGSEA).

Supplementary Table 7 22 kinds of infiltrating immune cell composition which calculate by CIBERSORT analysis.

Supplementary Table 8 Maximum inhibitory concentration (IC50) for cytotoxicity and Immunomodulator drugs pedicted in 884 cases of DLBCL.

Supplementary Table 9 The differentially biological pathways between cluster B and cluster A which obtained GSVA enrichment analysis.

Supplementary Table 10 Functional annotation and KEGG pathway for differentially expressed genes between cluster B and cluster A patients.
