# Supplementary figures and images for: Comprehensive Characterization of Cachexia-Inducing Factors in Diffuse Large B-Cell Lymphoma Reveals a Molecular Subtype and a Prognosis-Related Signature
Source: Front Cell Dev Biol. 2021 May 17;9:648856. doi: 10.3389/fcell.2021.648856 (PMC8166255; doi:10.3389/fcell.2021.648856)

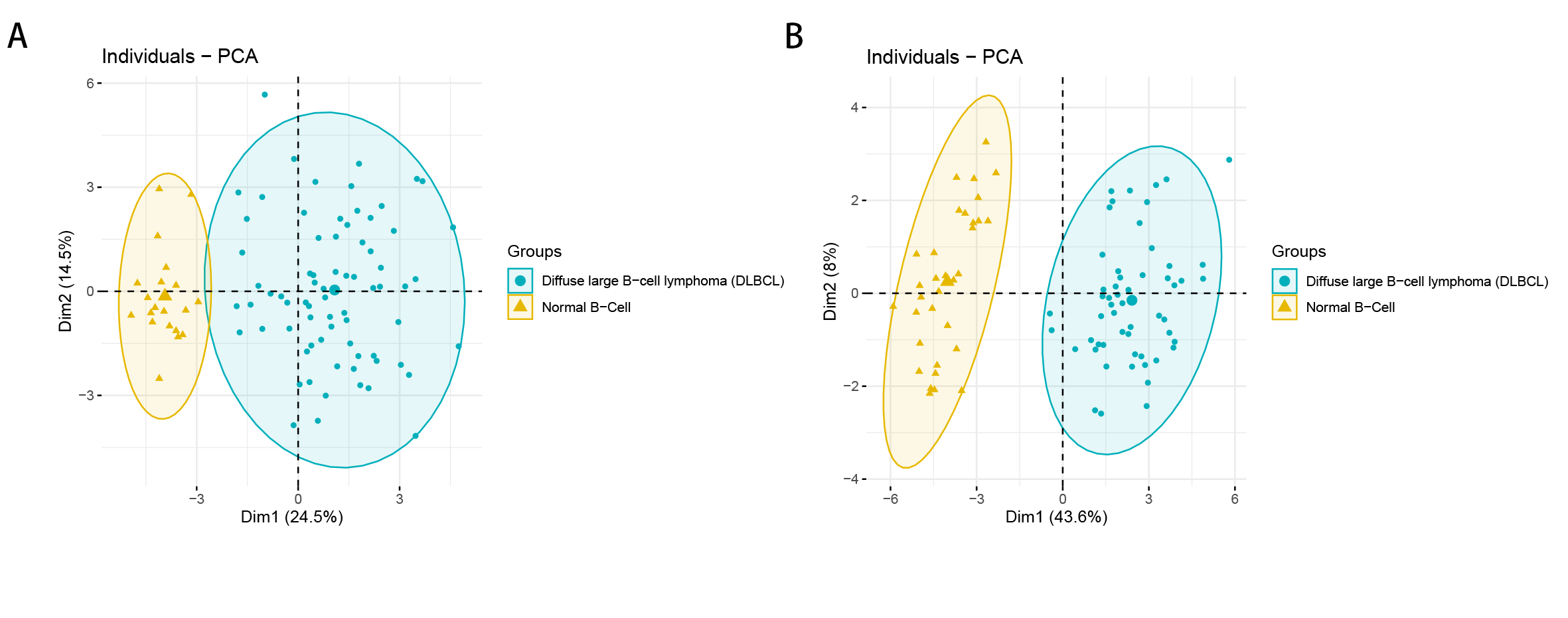

Supplement: Supplementary Figure 1 — Principal component analysis for the expression profiles of 25 cachexia-inducing factors (CIFS) to distinguish diffuse large B-cell lymphoma (DLBCL) from normal samples in GSE12195 and GSE56315 cohorts: (A,B). Two subgroups without intersection were identified, indicating the DLBCL, and normal samples were well distinguished based on the expression profiles of CIFs. [file Image_1.TIFF]

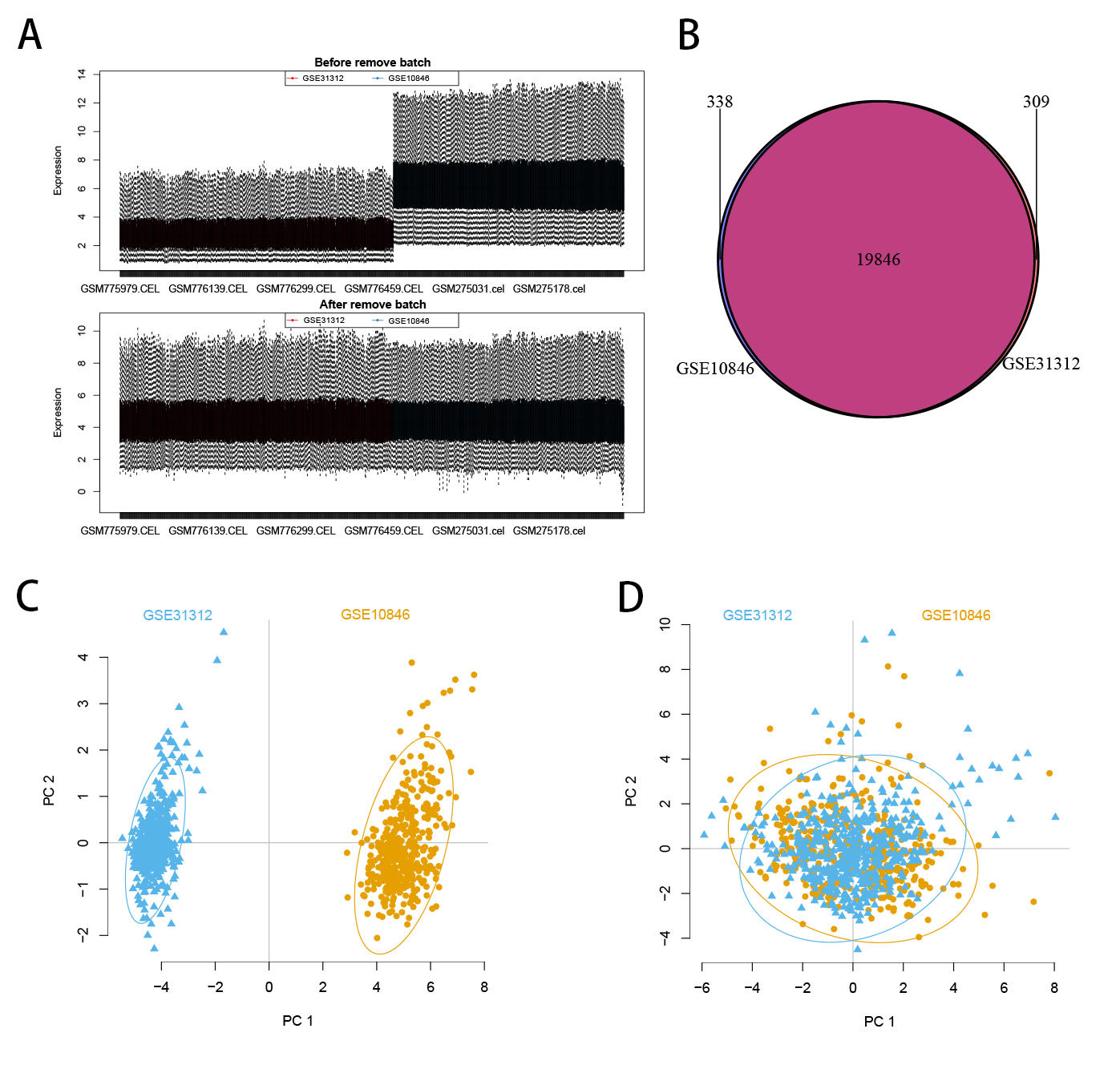

Supplement: Supplementary Figure 2 — Box plot of expression data before and after normalization. The x-axis presents the different cohorts, and the y-axis presents the expression value. (A) Data before and after normalization of the expression profiles of GSE10846 and GSE31312. (B) The Venn diagram for intersection of the probe set of GSE10846 and GSE31312. (C) Samples distribution of the two cohorts are significantly different before batch correction. (D) Samples distribution of the two cohorts after batch correction. [file Image_2.TIFF]

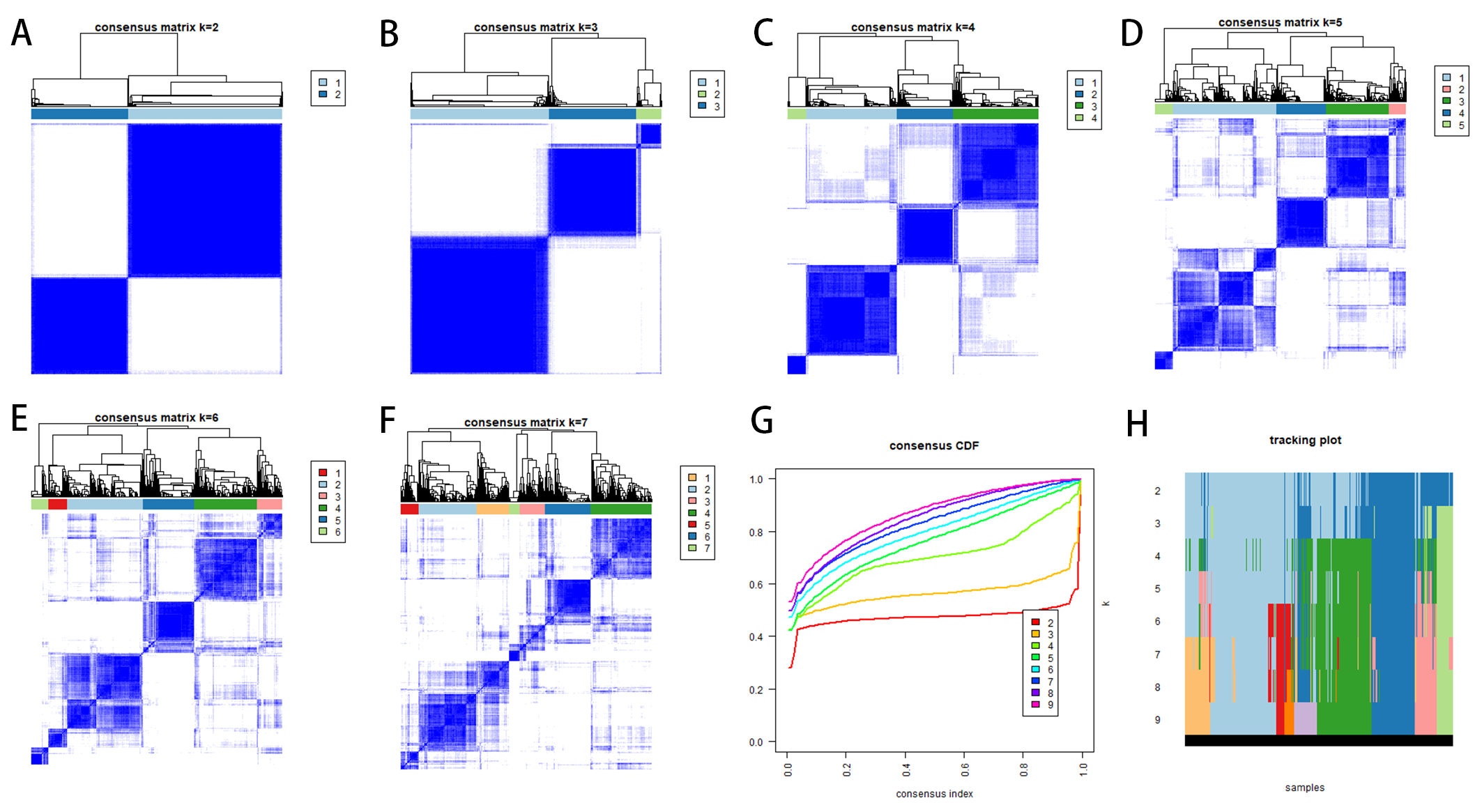

Supplement: Supplementary Figure 3 — Unsupervised clustering of 25 cachexia-inducing factors in 882 cases of patients with diffuse large B-cell lymphoma (DLBCL) to identify distinct molecular subtypes. (A–F) Consensus matrices of the DLBCL cohort for k = 2–7, allowing quick and accurate visualization of cluster boundaries. (G) Consensus clustering cumulative distribution function for k = 2 to 9. (H) Tracking plot showing the consensus cluster of items (in columns) at k = 2 to 9 (in rows). [file Image_3.TIFF]

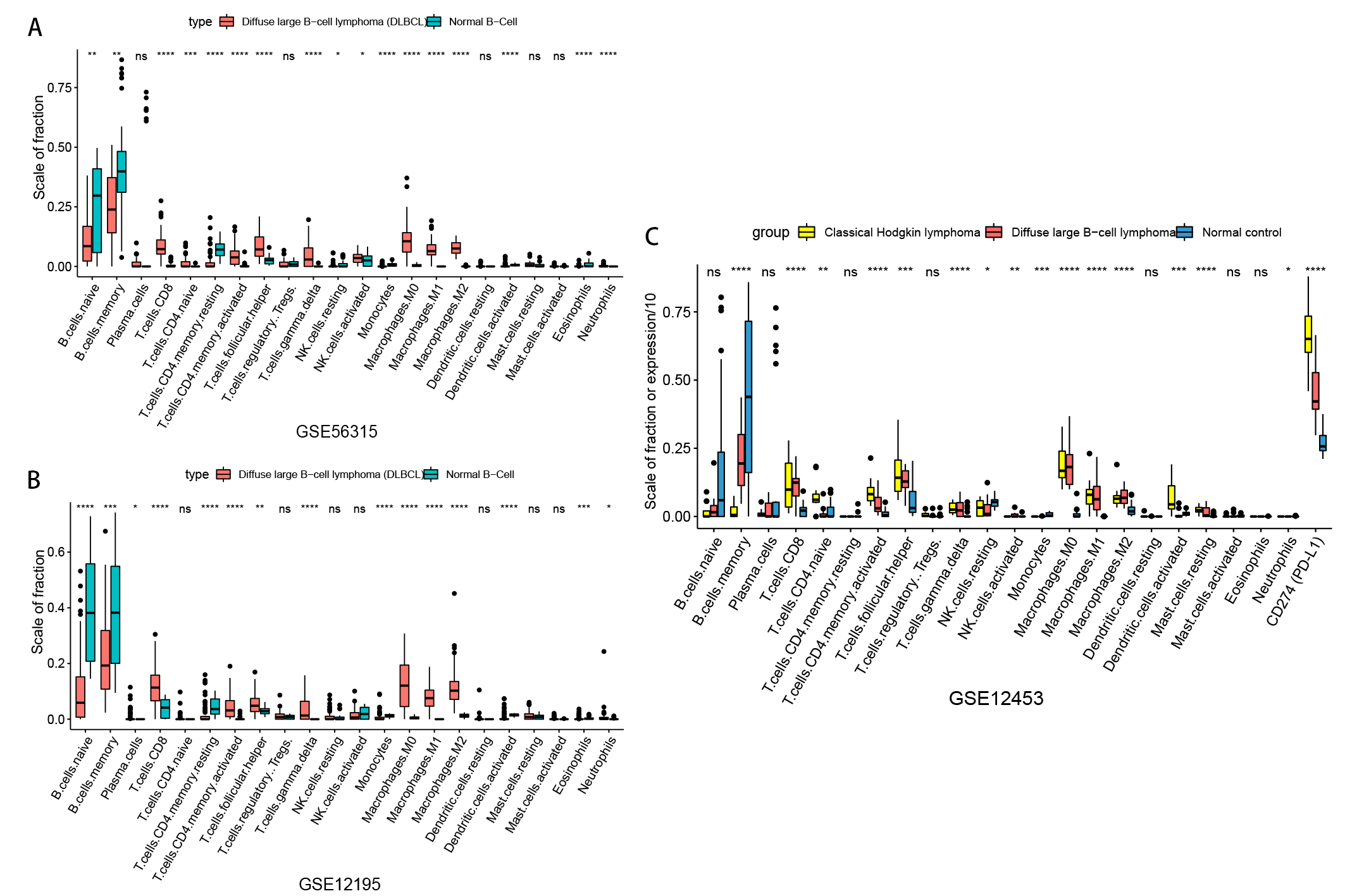

Supplement: Supplementary Figure 4 — Difference in the abundance of immune cell infiltration and expression of Pd-1 among diffuse large B-cell lymphoma (DLBCL), normal B cell, and classic Hodgkin’s lymphoma (cHl). (A,B) The proportion of immune cell in DLBCL and normal B cell extracted from tonsil: (A) GSE56315 and (B) GSE12195). (C) The proportion of immune cell infiltration and expression level of PD-L1 among DLBCL, normal B cell, and cHL. ∗P < 0.05; ∗∗P < 0.01; ∗∗∗P < 0.001, ****P < 0.0001. [file Image_4.TIFF]

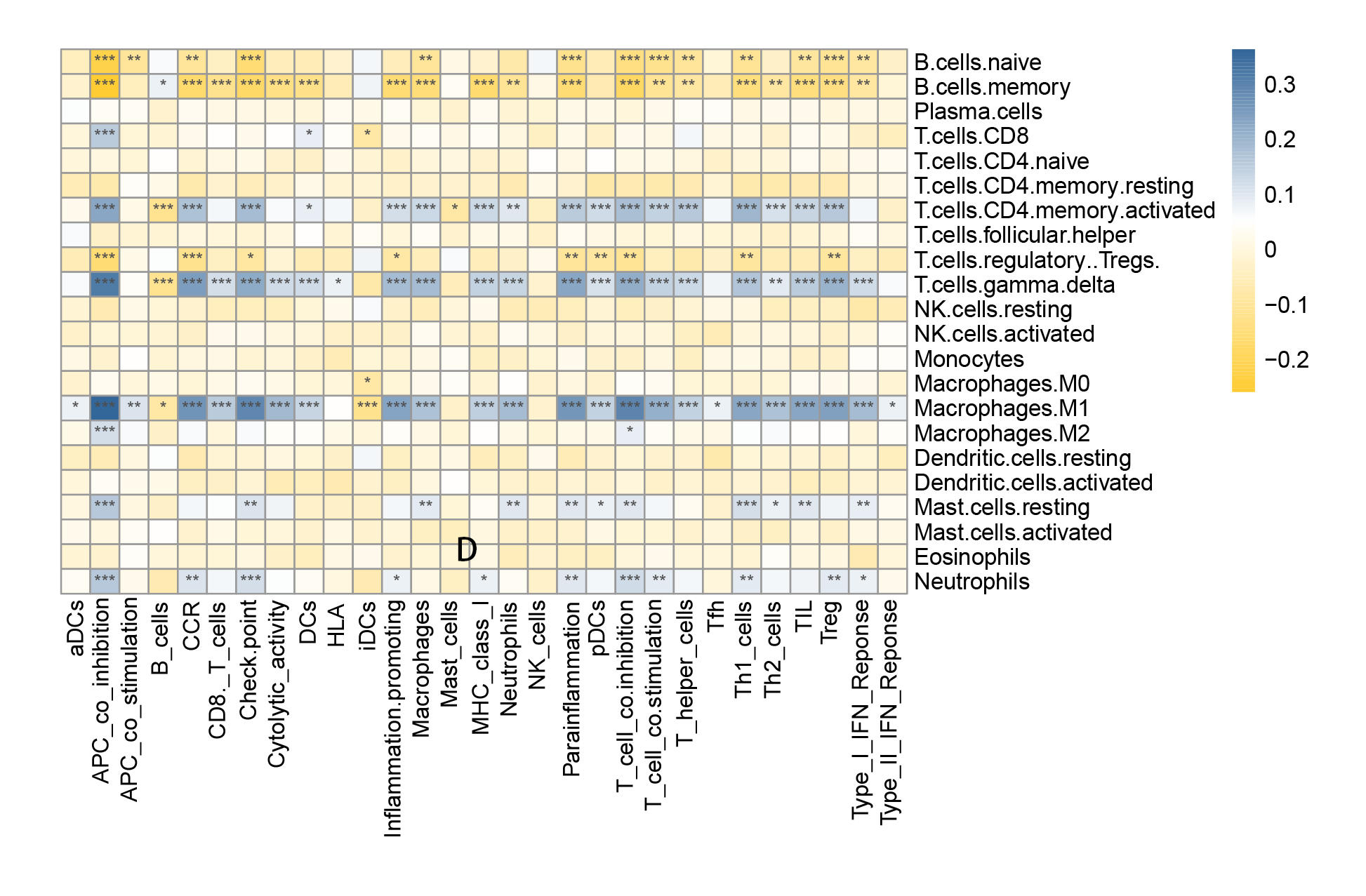

Supplement: Supplementary Figure 5 — Correlation of immune landscapes and immune cell infiltration. Positive correlation was marked with blue, and negative correlation was marked with yellow. [file Image_5.TIF]

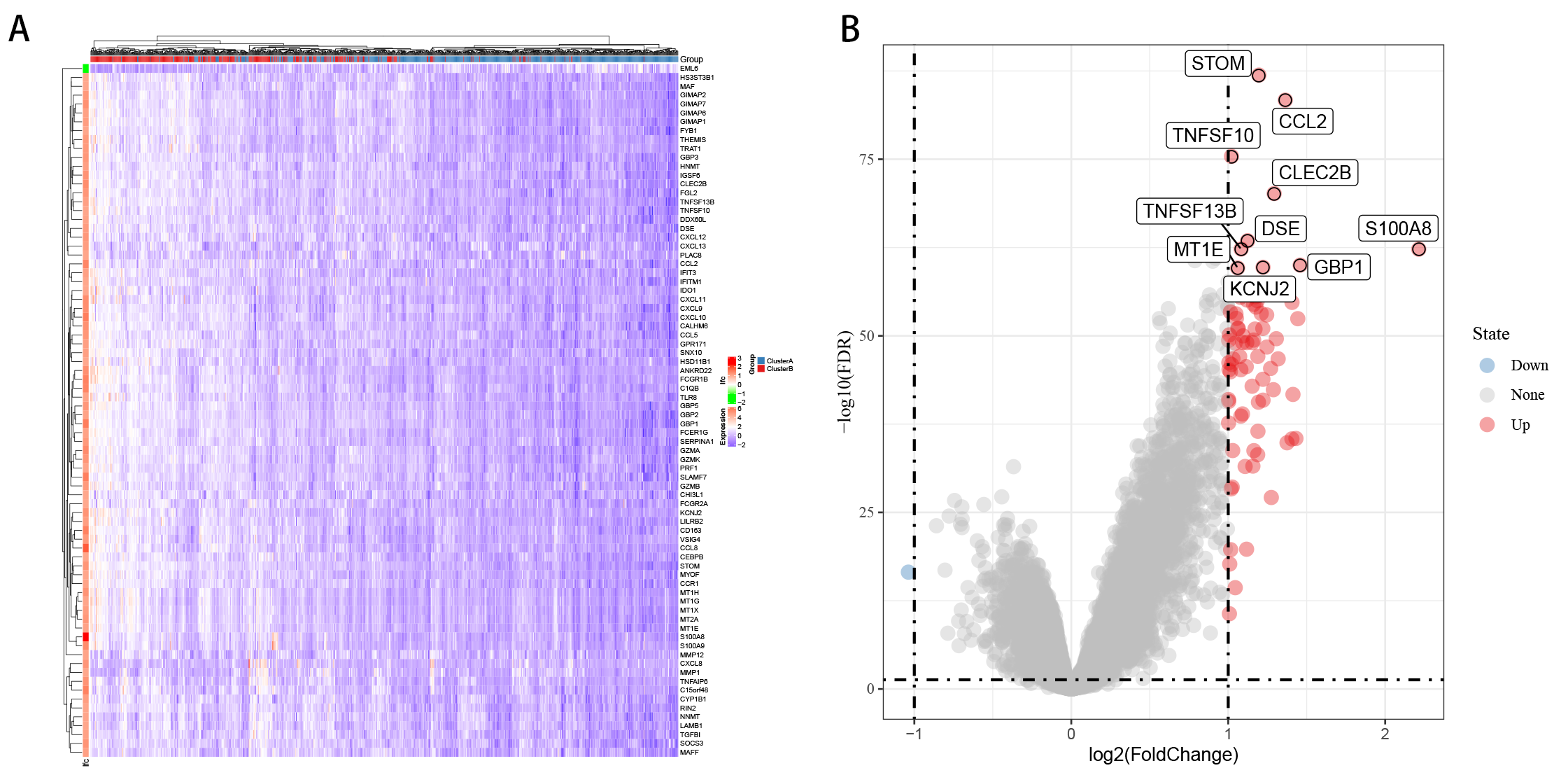

Supplement: Supplementary Figure 6 — Differentially expressed genes in cluster B and cluster a patients. (A) Heat map for differentially expressed genes in cluster B and cluster a patients. (B) Volcano plot of differentially expressed genes in cluster B and cluster a patients. Red, significantly upregulated genes; blue, significantly downregulated genes; Fc, fold change. [file Image_6.TIFF]

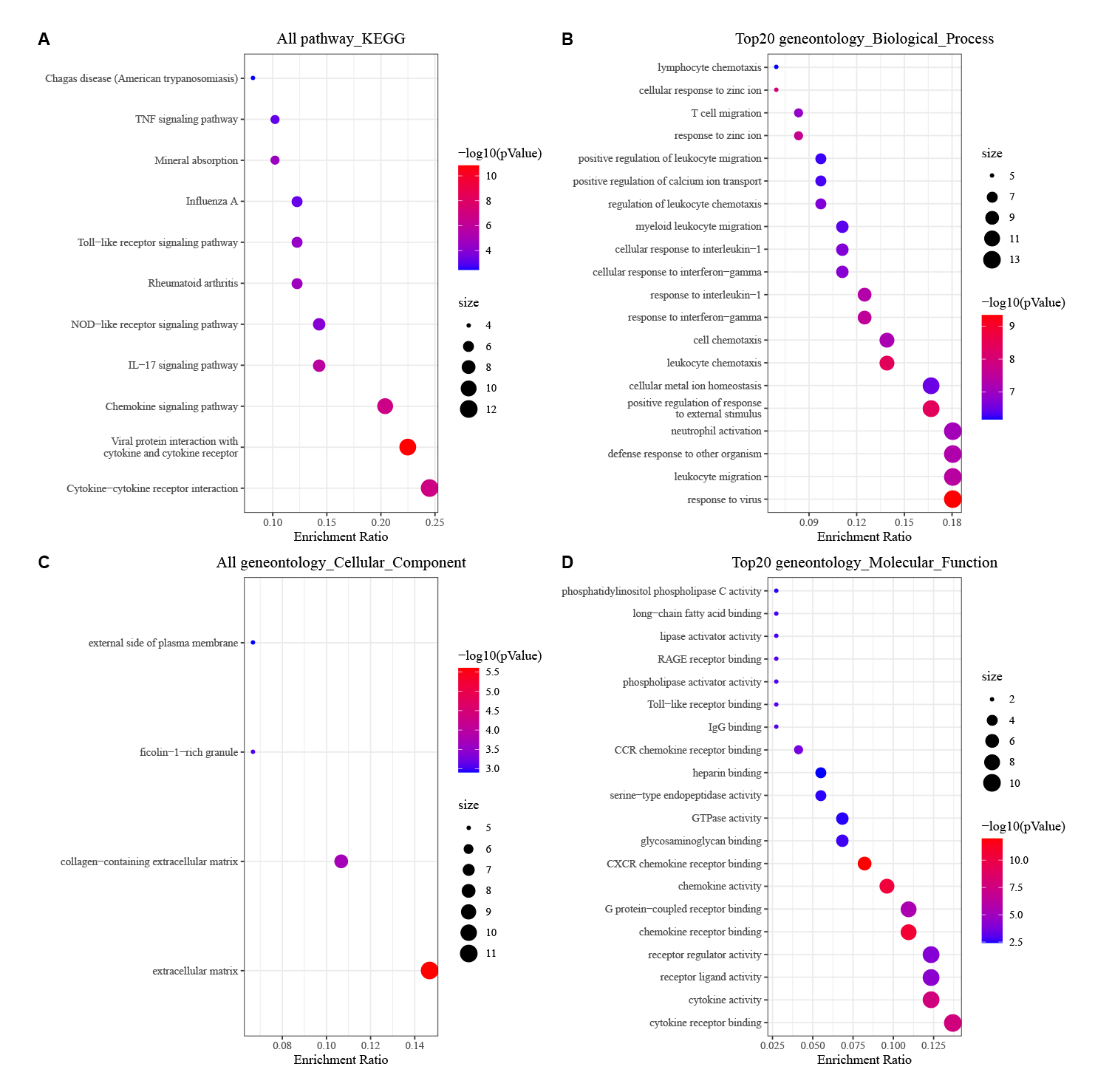

Supplement: Supplementary Figure 7 — Functional enrichment analysis for differentially expressed genes (DEGs) between cluster B and cluster a patients. (A) Kyoto Encyclopedia of Genes and Genomes analyses for DEGs. (B) Biological process. (C) Cellular component. (D) Molecular function. [file Image_7.TIFF]

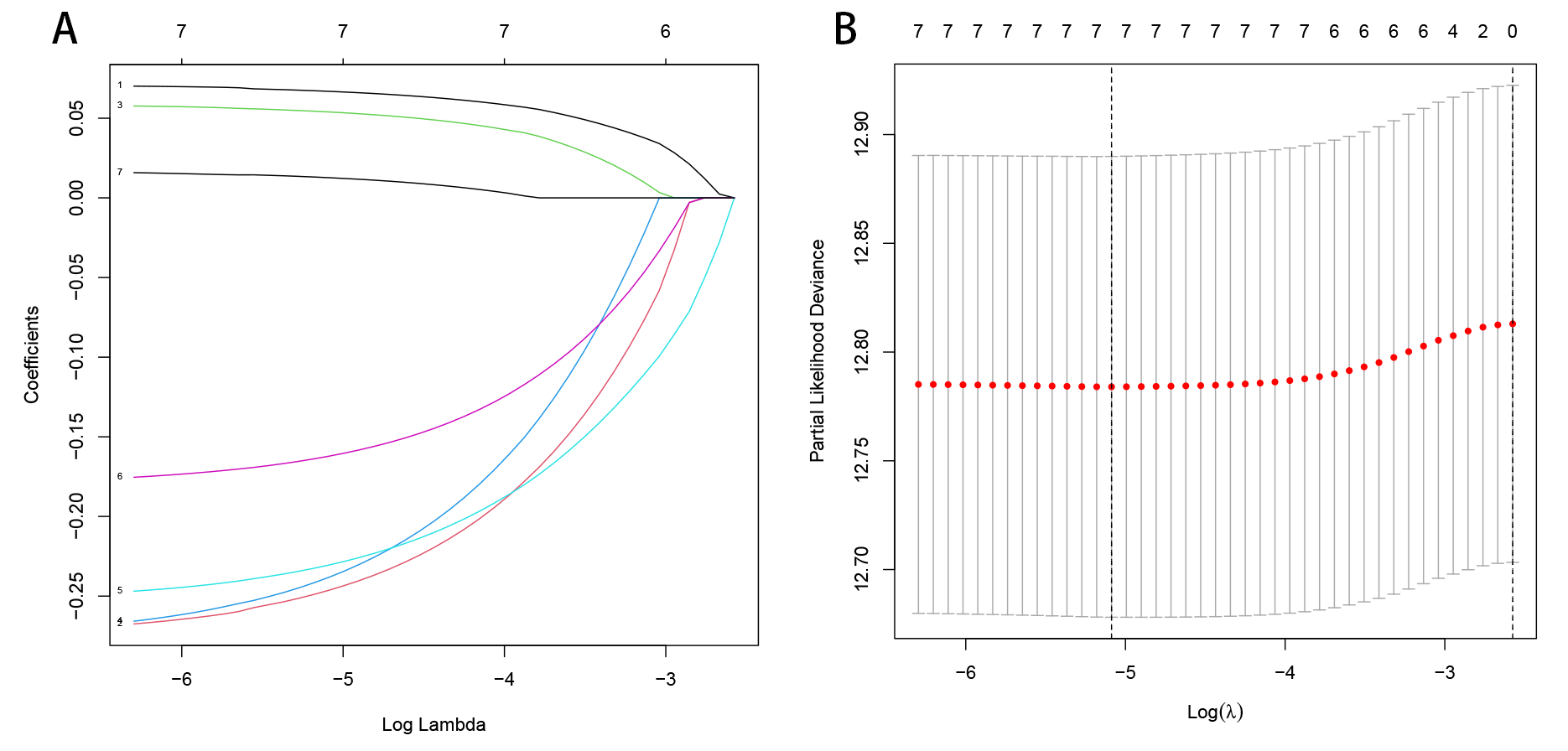

Supplement: Supplementary Figure 8 — Identification of the risk signature by least absolute shrinkage and selection operator (Lasso) Cox regression. (A) Lasso coefficient of the seven cachexia-inducing factors associated with overall survival in univariate Cox regression. (B) Ten-fold cross-validation for tuning the parameter selection in the Lasso module. [file Image_8.TIFF]

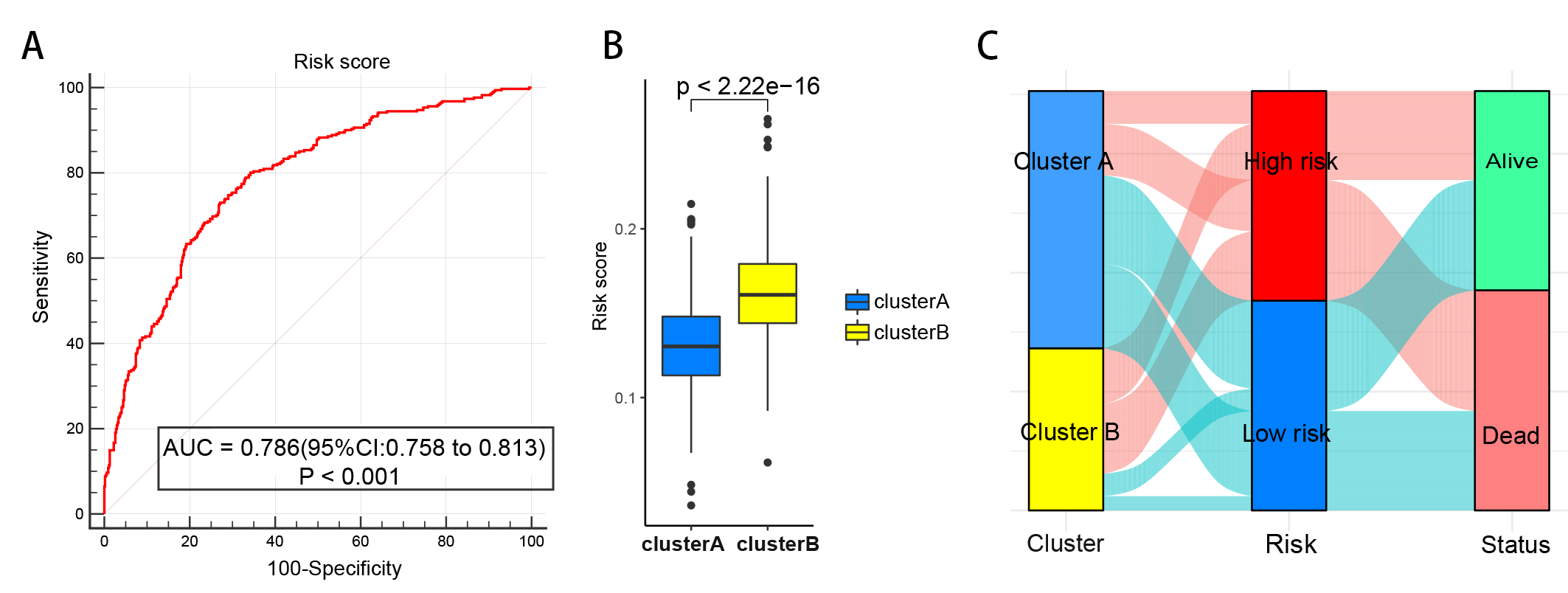

Supplement: Supplementary Figure 9 — Performance of the cachexia-inducing factor (CIFs) signature in identifying poor molecular subtypes in the training cohort. (A) Comparative risk score between cluster a subtype and cluster B subtype. (B) Receiver operating characteristic curves to depict the accuracy of CIFs risk signature in identifying cluster B which was with poor prognosis. (C) Alluvial diagram showing the changes of CIFs cluster subtypes, risk, and status. [file Image_9.TIFF]

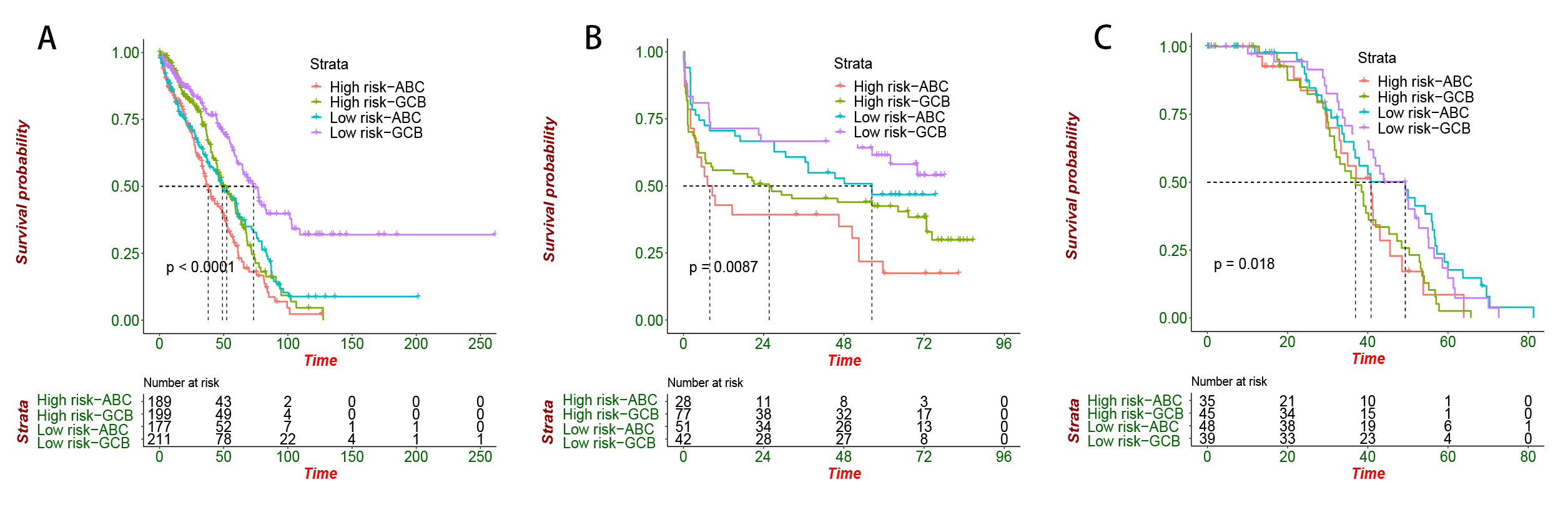

Supplement: Supplementary Figure 10 — Performance of combinations of the prognostic model and cell of origin subtype in the prediction of patients with DLBCL in the training cohort and two independent testing cohorts. (A) GSE10846 + GSE31312. (B) GSE87371. (C) GSE32918. [file Image_10.TIFF]
